# Development and validation of a nomogram for predicting subsyndromal delirium in critically ill patients in the emergency department

**DOI:** 10.1007/s11739-026-04448-1

**Published:** 2026-07-26

**Authors:** Changyun Wei, Hui Wang, Meng Su, Pengxia Sun, Jian Gao, Qin Zhu, Tianmei Feng, Tengyun Shang, Yunyi Le, Fan Li

**Affiliations:** 1 Department of Emergency, Peking Union Medical College Hospital, Chinese Academy of Medical Sciences and Peking Union Medical College, 41 Damucang Hutong, Xicheng District, Beijing, 100730 China; 2 Department of Endocrinology and Metabolism, Peking University Third Hospital, 49 North Garden Road, Haidian District, Beijing, 100191 China

**Keywords:** Subsyndromal delirium, Nomogram, Prediction model, Emergency department, Critically ill

## Abstract

This study aimed to develop and validate a nomogram for predicting subsyndromal delirium (SSD) risk in critically ill patients in the emergency department (ED). A retrospective cohort study was conducted on 712 patients admitted to the ED resuscitation room of Peking Union Medical College Hospital from January 2025 to August 2025. Patients were randomly divided into training (n = 525) and validation (n = 187) sets. Multivariate logistic regression analyses were performed to identify independent risk factors for SSD. A nomogram prediction model was constructed based on these factors and evaluated using receiver operating characteristic (ROC) curves, calibration plots, and decision curve analysis (DCA). Multivariate analysis identified six independent predictors of SSD: marital status (divorced: OR = 12.210, 95%CI 1.334–111.750), physical restraint (OR = 10.386, 95%CI 2.932–36.786), RASS score (OR = 4.090, 95%CI 2.029–8.243), APACHE II score (OR = 1.092, 95%CI 1.011–1.180), Barthel index (OR = 0.968, 95%CI 0.951–0.984), and PSQI score (OR = 1.239, 95%CI 1.123–1.368). The nomogram demonstrated excellent discrimination, with an AUC of 0.898. The calibration curve showed good agreement between predicted and actual probabilities. DCA indicated that the nomogram had significant clinical net benefit across a wide range of threshold probabilities. This study developed a novel nomogram that incorporates six readily available clinical variables. This tool effectively predicts the individual risk of SSD in ED critically ill patients, facilitating early identification and targeted preventive interventions to improve patient outcomes.

## Introduction

In critically ill patients, delirium is a frequent and serious complication. This acute neuropsychiatric syndrome is characterized by fluctuating disturbances in attention, awareness, and cognition [1, 2]. Among patients aged 65 and older presenting to the emergency department, the reported incidence of delirium is approximately 10%, underscoring it as a common and serious concern, especially for the elderly and critically ill [3]. The condition is strongly associated with several serious adverse outcomes: extended hospital stays, accelerated cognitive decline, increased mortality, and greater healthcare expenditures [4].

In recent years, increasing attention has been paid to subsyndromal delirium (SSD), a clinical state where patients exhibit one or more symptoms of delirium but do not meet the full diagnostic criteria [5, 6]. As an intermediate state between normal cognition and delirium, subsyndromal delirium (SSD) is associated with similarly adverse outcomes, including cognitive impairment, prolonged mechanical ventilation and ICU hospitalization, and an increased risk of progression to full delirium and death [7, 8]. With a reported prevalence ranging from 11.7% to 69% among critically ill patients, SSD represents a highly prevalent and major clinical concern in the emergency department (ED) [9].

The ED resuscitation room represents a high-risk setting for SSD among critically ill patients. This is due to a combination of factors including acute physiological stress, comorbidities, environmental triggers like noise and sleep disruption, along with frequent invasive procedures and restraint use [10]. While several risk factors for SSD have been identified, such as advanced age, cognitive impairment, severity of illness, and sleep deprivation, a practical, individualized tool for predicting SSD risk upon ED admission is currently lacking [11].

Nomograms are visual tools used in clinical assessment. They represent predictive models graphically, allowing clinicians to calculate a patient’s risk score by summing points assigned to key factors [12]. This study aimed to investigate the prevalence of SSD in a Chinese cohort of ED critically ill patients, identify independent risk factors, and subsequently develop and validate a clinically useful nomogram for the early prediction of SSD.

## Methods

### Study design and participants

This single-center, retrospective cohort study was conducted at the ED resuscitation room of Peking Union Medical College Hospital. The Institutional Review Board of Peking Union Medical College Hospital approved this study. Informed consent was waived due to its retrospective design. A total of 712 patients admitted from January 2025 to August 2025 were enrolled and randomly assigned to a training set (n = 525) or a validation set (n = 187). Patients were selected based on the following criteria. Inclusion criteria: (1) age ≥ 18 years; (2) ED resuscitation room stay > 24 h; (3) no history of psychotropic drug addiction; (4) not being in a post-general-anesthesia state; (5) Glasgow coma scale (GCS) score ≥ 13 at admission. Exclusion Criteria: (1) pre-existing psychiatric disorders diagnosed according to the Diagnostic and Statistical Manual of Mental Disorders, Fifth Edition; (2) being in a state of deep sedation (RASS score between −5 and −3); (3) the presence of delirium at admission or progression from SSD to delirium during the stay; (4) CT-confirmed organic brain injury.

### Data collection and definitions

Data were extracted from the hospital's electronic medical records by trained researchers. The demographics and clinical characteristics included age, sex, education level, marital status, payment source, comorbidities (hypertension, diabetes, coronary heart disease), smoking history, drinking history, surgery history, transfusion history, fever at admission (> 37.3 °C), use of physical restraint, and mechanical ventilation. All assessment scales were assessed within 24 h of admission: (1) pain: assessed using the Numerical Rating Scale (NRS), ranging from 0 (no pain) to 10 (worst pain imaginable) [13]; (2) sedation level: assessed using the Richmond Agitation-Sedation Scale (RASS), [14] which ranges from −5 (unarousable) to + 4 (combative); (3) disease severity: assessed using the Acute Physiology and Chronic Health Evaluation II (APACHE II) score [15], with higher scores indicating greater severity; (4) activities of daily living: assessed using the Barthel Index [16], which ranges from 0 (completely dependent) to 100 (completely independent); (5) frailty: assessed using the FRAIL scale [17], with a score of 0 indicates robustness, 1–2 pre-frailty, and ≥ 3 frailty; (6) sleep quality: assessed using the Pittsburgh Sleep Quality Index (PSQI) [18], with a global PSQI score > 8 indicates poor sleep quality.

### SSD diagnosis

SSD was diagnosed using the Intensive Care Delirium Screening Checklist (ICDSC) [19]. The ICDS is an eight-item screening tool assessing the following domains: altered level of consciousness, inattention, disorientation, hallucination/delusion/psychosis, psychomotor agitation or retardation, inappropriate speech or mood, sleep–wake cycle disturbance, and symptom fluctuation. A total score of 1–3 was defined as SSD, while a score of ≥ 4 indicates delirium. Independent assessments were carried out by two charge nurses to ensure reliability. The deputy chief nurse was responsible for making the final judgment in cases of disagreement.

### Development and validation of nomogram

The nomogram was constructed based on the results of the multivariate logistic regression model using the rms package in R software (version 4.2.2). Discrimination and calibration of the nomogram were evaluated separately in the training set and the validation set. The performance of nomogram was evaluated by using two complementary metrics. First, discrimination (the model’s ability to correctly differentiate between patients who will develop SSD and those who will not) was measured by the area under the curve (AUC). Second, calibration (the degree of agreement between the predicted probability of SSD and the actual observed frequency of SSD) was assessed using a calibration plot. To obtain more stable and reliable estimates of calibration and to minimize the risk of overfitting, we applied 1000 bootstrap resamples. To evaluate the clinical utility of the nomogram, decision curve analysis (DCA) was conducted, which quantifies the net benefit across a range of threshold probabilities.

### Statistical analysis

Continuous variables were presented as mean ± standard deviation (SD) or median (interquartile range, IQR) and compared using Student’s *t*-test or Mann–Whitney *U* test, as appropriate. Categorical variables were presented as numbers (percentages) and compared using the Chi-square test or Fisher’s exact test. Variables were included in the multivariate binary logistic regression analysis using the backward stepwise method to identify independent risk factors for SSD. Odds ratio (OR) and 95% confidence interval (CI) were calculated. A two-sided *P*-value < 0.05 was considered statistically significant. Statistical analyses were performed using SPSS 25.0.

## Results

### Patient characteristics and prevalence of SSD

A total of 525 patients were included in the training set. Among them, 156 (29.7%) were diagnosed with SSD during their ED stay. The baseline characteristics of the two groups are summarized in Table 1. Compared to the non-SSD group, patients in the SSD group were significantly older (63.0 ± 16.0 *vs.* 56.8 ± 16.6 years, *P* < 0.001), had a higher prevalence of hypertension (51.9% *vs.* 41.5%, *P* = 0.028) and diabetes (33.3% vs. 22.8%, *P* = 0.012), but a lower prevalence of drinking history (21.2% *vs.* 30.4%, *P* = 0.031). Furthermore, the SSD group had significantly higher rates of physical restraint (34.6% *vs.* 1.4%, *P* < 0.001) and fever at admission (29.5% *vs.* 18.4%, *P* = 0.005). Regarding assessment scores, the SSD group had significantly higher RASS scores, APACHE II scores, FRAIL scores, and PSQI scores, but lower Barthel Index scores (all *P* < 0.001).

**Table 1:** Demographic data and clinical characteristics of training set

Characteristics	SSD (n = 156)	non-SSD (n = 369)	t/Z/χ^2^ values	*P* values
Age, years	63.0 ± 16.0	56.8 ± 16.6	− 3.923	< 0.001
Sex (n, %)			2.777	0.096
male	79 (49.4)	216 (58.5)		
female	77 (50.6)	153 (41.5)		
Education level (n, %)			23.394	0.001
illiterate	11 (7.1)	10 (2.7)		
primary school	35 (22.4)	42 (11.4)		
junior high school	42 (26.9)	96 (26.0)		
senior high school	35 (22.4)	93 (25.2)		
Bachelor’s degree	31 (19.9)	111 (30.1)		
postgraduate	2 (1.3)	17 (4.6)		
Marital status (n, %)			4.411	0.110
unmarried	7 (4.5)	35 (9.5)		
married	144 (92.3)	327 (88.6)		
divorced	5 (3.2)	7 (1.9)		
Payment source (n, %)			0.224	0.636
self-pay	28 (17.9)	60 (16.3)		
medical insurance	128 (82.1)	309 (83.7)		
Hypertension (n, %)	81 (51.9)	153 (41.5)	4.856	0.028
Diabetes (n, %)	52 (33.3)	84 (22.8)	6.381	0.012
Coronary heart disease (n, %)	27 (17.3)	61 (16.5)	0.047	0.828
Smoking history (n, %)	46 (29.5)	118 (32.0)	0.371	0.574
Drinking history (n, %)	33 (21.2)	112 (30.4)	4.641	0.031
Surgery history (n, %)	9 (5.8)	37 (10.0)	2.497	0.115
Transfusion history (n, %)	21 (13.5)	45 (12.2)	0.160	0.689
Fever at admission (n, %)	46 (29.5)	68 (18.4)	7.889	0.005
Physical restraint (n, %)	54 (34.6)	5 (1.4)	121.598	< 0.001
Mechanical ventilation (n, %)	9 (5.8)	10 (2.7)	2.942	0086
NRS score	1.0 (1.0, 2.0)	1.0 (1.0, 2.0)	4.189	< 0.001
RASS score	1.0 (0, 1.0)	0 (0, 0)	12.881	< 0.001
APACHEⅡ score	11.0 (8.0, 13.9)	8.0 (6.0, 10.0)	7.283	< 0.001
Barthel index	60 (50, 74.5)	95 (85, 100)	− 12.004	< 0.001
FRAIL score	2.0 (1.0, 2.0)	1.0 (1.0, 1.0)	8.506	< 0.001
PSQI score	9.0 (7.0, 11.0)	6 (4.0, 7.0)	9.476	< 0.001

Data are presented as mean ± SD, number (%) or median (interquartile range), as appropriate

### Variable selection and multivariate logistic regression analysis

The results of the multivariate logistic regression analysis are shown in Table 2. Six variables were identified as independent predictors of SSD. Being divorced (OR = 12.210, 95% CI 1.334–111.750, *P* = 0.027) was associated with a higher risk of SSD compared to being unmarried. Physical restraint (OR = 10.386, 95% CI 2.932–36.786, *P* < 0.001), higher APACHE II Score (OR = 1.092, 95% CI 1.011–1.180, *P* = 0.025), PSQI Score (OR = 1.239, 95% CI 1.123–1.368, *P* < 0.001) and RASS score (OR = 4.090, 95% CI 2.029–8.243, *P* < 0.001) were associated with a higher risk of SSD, while higher Barthel Index (OR = 0.968, 95% CI 0.951–0.984, *P* < 0.001) indicated a lower risk of SSD.

**Table 2:** Multivariate logistic regression for the baseline clinical characteristics associated with SSD

Characteristics	B	Wald	*P* values	Exp (B)	95% CI	
					Lower limit	Upper limit
Age	0.004	0.075	0.785	1.004	0.978	1.030
Sex-male	0.155	0.152	0.697	1.168	0.535	2.548
Education level (illiterate)		2.396	0.792			
primary school	−0.883	1.165	0.280	0.413	0.083	2.056
junior high school	−0.795	0.996	0.318	0.468	0.105	2.079
senior high school	−0.825	1.130	0.288	0.438	0.096	2.006
bachelor's degree	−0.454	0.774	0.345	0.635	0.139	2.893
postgraduate						
Marital status (unmarried)		6.246	0.044			
married	0.665	0.535	0.465	1.945	0.327	11.568
divorced	2.502	4.907	0.027	12.210	1.334	111.750
Payment source (self-pay)	−0.641	2.122	0.145	0.527	0.222	1.248
Hypertension (n, %)	0.283	0.638	0.424	1.327	0.663	2.657
Diabetes (n, %)	0.077	0.042	0.837	1.080	0.520	2.244
Coronary heart disease (n, %)	−0.235	0.292	0.589	0.791	0.337	1.855
Smoking history (n, %)	0.899	3.657	0.056	2.458	0.978	6.176
Drinking history (n, %)	−0.640	2.047	0.153	0.527	0.219	1.267
Surgery history (n, %)	−0.654	0.735	0.391	0.520	0.117	2.318
Transfusion history (n, %)	−0.109	1.946	0.822	1.734	0.800	3.760
Fever at admission (n, %)	0.551	0.051	0.163	0.897	0.346	2.322
Physical restraint (n, %)	2.340	13.158	< 0.001	10.386	2.932	36.786
Mechanical ventilation (n, %)	−0.356	0.195	0.659	0.700	0.144	3.399
NRS score	0.409	3.359	0.067	1.505	0.972	2.330
RASS score	1.409	15.515	< 0.001	4.090	2.029	8.243
APACHEⅡ score	0.088	5.035	0.025	1.092	1.011	1.180
Barthel index	−0.033	14.071	< 0.001	0.968	0.951	0.984
FRAIL score	0.073	0.061	0.805	1.075	0.604	1.915
PSQI score	0.215	18.171	< 0.001	1.239	1.123	1.368

### Development and presentation of the nomogram

**Fig. 1:**
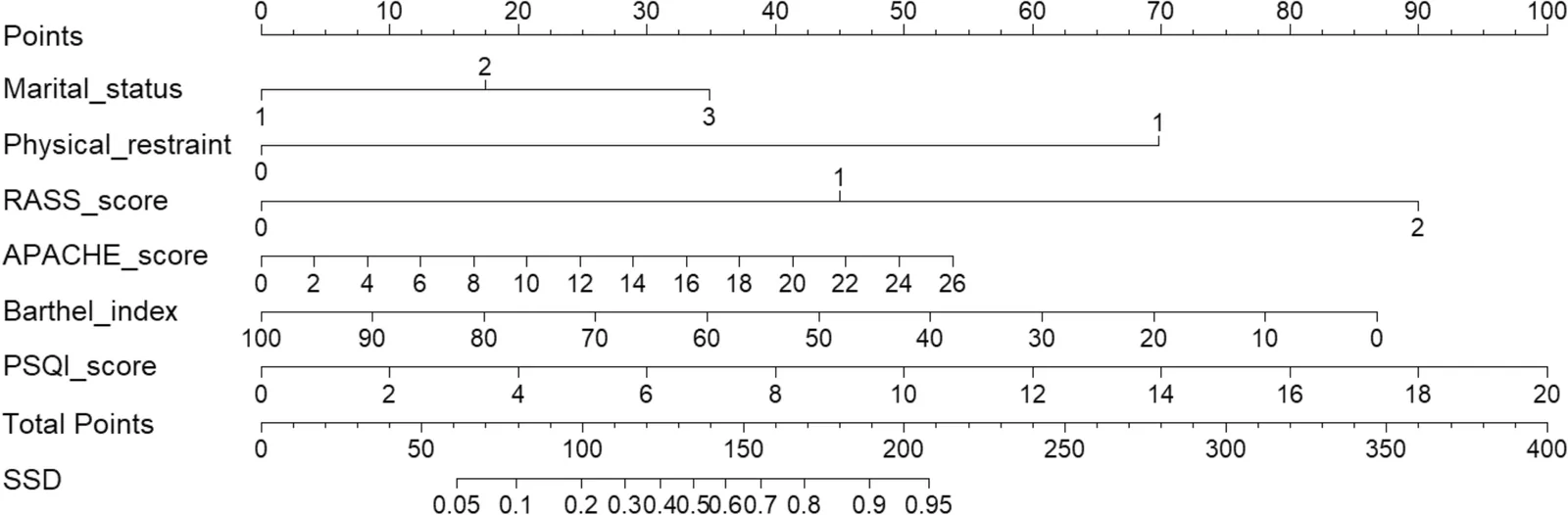
Nomogram to predict the probability of SSD in critically ill patients. Note: Marital status: 1 = unmarried, 2 = married, 3 = divorced; Physical restraint: 0 = non-physical restraint, 1 = physical restraint

Based on the multivariate logistic regression model, a nomogram was constructed to predict the individual risk of SSD (Fig. 1). Clinicians can determine an individual's SSD risk using the nomogram in three steps: 1) for each variable, find its corresponding score on the “Points” axis; 2) sum all scores and locate the value on the “Total Points” axis; 3) read the predicted probability directly below this total on the “Risk of SSD” axis.

### Validation of the nomogram

**Fig. 2:**
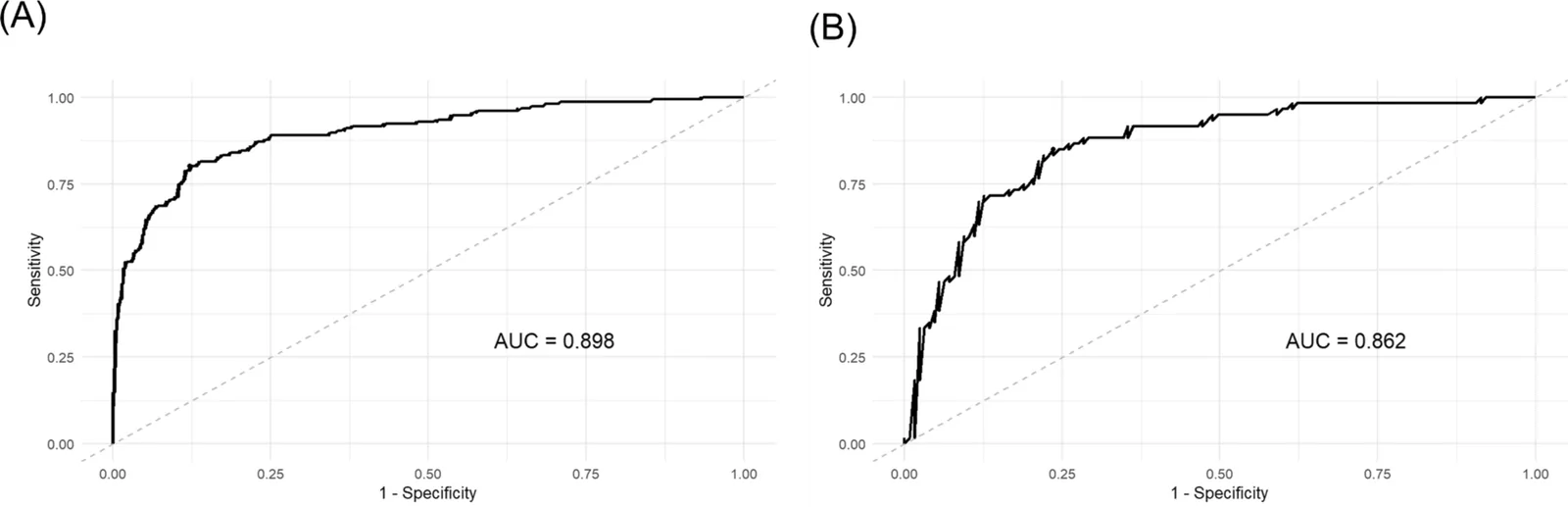
ROC curves of the nomogram in the training group (**A**) and testing group (**B**)

**Fig. 3:**
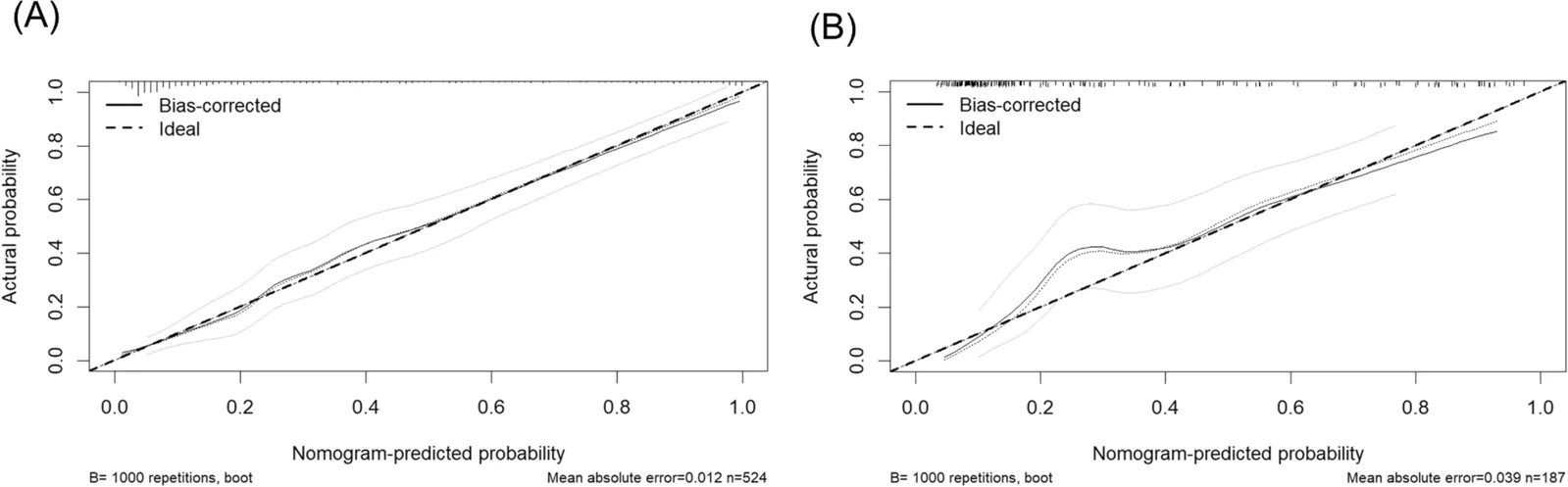
Calibration plots of the nomogram in the training group (**A**) and testing group (**B**)

**Fig. 4:**
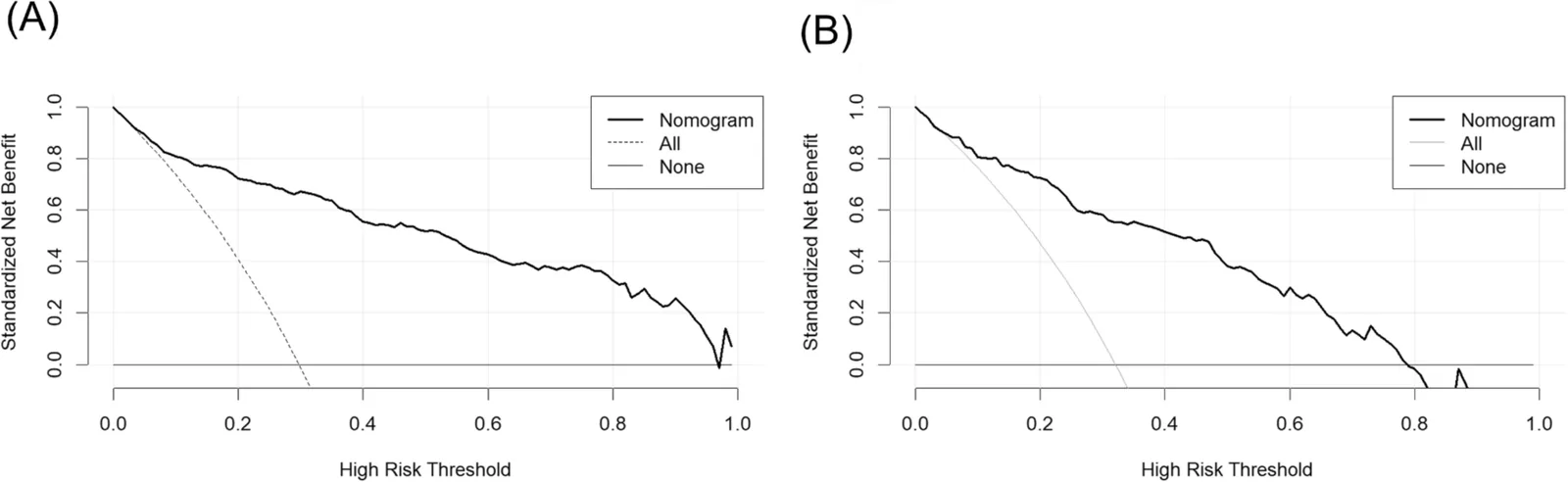
Decision curves of the nomogram in the training group (**A**) and testing group (**B**)

The nomogram demonstrated excellent discriminatory power, with an AUC of 0.898 (95% CI 0.867–0.929) in training set (Fig. 2A) and 0.862 (95% CI 0.806–0.918) in validation set. The calibration plot showed good consistency between the nomogram-predicted probability and the actual observed probability of SSD both in training set (Fig. 3A) and validation set (Fig. 3B). Decision curve analysis revealed that using the nomogram to predict SSD risk provided a positive net benefit across a wide range of threshold probabilities, demonstrating its clinical utility both in training set and validation set (Fig. 4).

## Discussion

This study developed and validated a novel nomogram for predicting the risk of SSD in critically ill patients admitted to the ED resuscitation room. The model incorporates six easily obtainable clinical variables assessed at admission: marital status, physical restraint, RASS score, APACHE II score, Barthel Index, and PSQI score. The nomogram exhibited excellent discrimination, good calibration, and favorable clinical utility, suggesting it can serve as a practical tool for early risk stratification.

Our study identified a substantial SSD prevalence of 29.7% in the training cohort of critically ill patients admitted to the ED resuscitation room. This figure occupies an intermediate position within the broad prevalence range (11.7%−69%) reported in previous literature for critical care settings [9]. The relatively high prevalence observed in our cohort highlights SSD as a common yet under‑recognized entity in emergency critical care. This finding is closely associated with the high‑acuity profile of our study population—patients requiring resuscitation care, who are inherently exposed to greater acute physiological stressors and environmental triggers.

The strong association between higher RASS scores (agitation) and SSD risk is consistent with the hyperactive and mixed subtypes of delirium [20]. Agitation can be an early manifestation of underlying cerebral dysfunction. The APACHE II score, a marker of overall physiological derangement and illness severity, is a well-established risk factor for delirium and its subsyndromal forms [21, 22]. Our findings confirm that sicker patients are more vulnerable to SSD.

The protective effect of a higher Barthel Index underscores the importance of functional status. Patients with greater independence in daily activities likely have better cognitive reserve and physical resilience, which may buffer against the acute insults that precipitate SSD [23]. Similarly, poor sleep quality, as measured by the PSQI, is a potent and modifiable risk factor. Sleep–wake cycle disruption is a core feature of delirium, and the ED environment, with its constant lighting and noise, severely compromises sleep, potentially triggering a cascade of neuroinflammatory processes involved in SSD pathogenesis [24].

The strong association between divorced marital status and elevated SSD risk is a key finding. While univariate analysis showed no group difference, the multivariate model—after adjusting for confounders like age and comorbidities—revealed this robust link. This suggests the effect is independent of other demographic factors, pointing to divorced individuals as a distinct population with specific psychosocial vulnerabilities in critical illness. This chronic stress may erode psychological resilience, rendering these individuals more susceptible to acute cognitive dysfunction under the additional, profound stress of critical illness and the disorienting ED environment [25]. The absence of an immediate, familiar support person at the bedside may further exacerbate feelings of isolation and disorientation.

The identification of physical restraint as a major independent risk factor for SSD is consistent with a substantial body of literature linking restraint to delirium spectrum disorders [26]. The pathophysiological link between physical restraint and SSD likely involves two interrelated mechanisms. On one hand, restraint can induce intense psychological distress (fear, anxiety, and helplessness) that may directly precipitate cognitive disturbances. On the other hand, it imposes severe sensory and motor deprivation, disrupting sensory processing and the sleep‑wake cycle, both of which are established pathways to delirium [24]. It is also plausible that restraint use and SSD share a bidirectional relationship: agitation from emerging SSD may prompt restraint application, which in turn exacerbates the cognitive dysfunction, creating a vicious cycle.

Unlike a study that focused on SSD of among elderly patients with pancreatic surgery [27], education level showed no significant association with SSD risk in our multivariate analysis. This finding suggests that in the acute ED setting, the protective effects of higher education on cognitive reserve may be overwhelmed by acute physiological stressors. As age increases, patients suffered from decline in neurologic and immune system function and it has been reported that age is an independent risk factor for SSD [28, 29]. However, in our present study, age demonstrated non-significant associations in the multivariate model and this indicates that their effects may be mediated through other variables such as functional status or disease severity, as well as increasing comorbidities.

Several limitations should be acknowledged. First, this was a single-center retrospective study, which may limit the generalizability of our findings. Second, the diagnosis of SSD was based on the ICDSC. While this instrument is validated, its application has two inherent constraints: it depends on consistent nursing evaluations, and it may be influenced by inter-rater variation, even with a protocol to resolve disagreements. Third, the model requires external validation in prospective, multi-center cohorts to confirm its robustness and transportability before widespread clinical implementation. At last, it should be noted that the odds ratio for the ‘divorced’ category has a wide confidence interval, indicating some statistical instability likely due to the small number of patients in this subgroup.

## Conclusions

In conclusion, we have developed a user-friendly nomogram that effectively predicts the risk of SSD in critically ill ED patients. This tool integrates six clinical variables readily available at admission. The nomogram has the potential to guide the timely implementation of preventive bundles aimed at mitigating the burden of SSD, potentially preventing its progression to delirium, and improving overall patient outcomes.

## Data Availability

The datasets used and/or analyzed during the current study are available from the corresponding author upon reasonable request.
